# Coordinated interactions among Nipah virus N, P and M proteins drive formation of distinct inclusion bodies

**DOI:** 10.1099/jgv.0.002206

**Published:** 2025-12-17

**Authors:** Mitsuki Yasukochi, Mika Hosogi, Yuki Kitai, Yukiko Akahori, Akihide Ryo, Makoto Takeda, Hiroshi Katoh

**Affiliations:** 1 Department of Microbiology, Graduate School of Medicine and Faculty of Medicine, The University of Tokyo, Tokyo 113-0033, Japan; 2 Department of Bioinformatics and Integrative Omics, National Institute of Infectious Diseases, Japan Institute for Health Security, Tokyo 162-8640, Japan

**Keywords:** inclusion body, membrane-less organelle property, Nipah virus, viral protein interactions

## Abstract

Nipah virus (NiV), a highly pathogenic zoonotic paramyxovirus, forms two distinct types of membrane-less organelles called inclusion bodies (IBs): cytosolic IBs, which serve as sites of viral RNA synthesis, and those beneath the plasma membrane (IB-PMs), which function in viral particle assembly and budding. We identified the essential domains of the NiV nucleocapsid (N) and phospho (P) proteins required for the formation of cytosolic IB-like structures with liquid-like properties. Dual-site interactions between the N- and C-terminal regions of the N and P proteins were necessary for generating these liquid organelles. In contrast, the matrix protein, along with the N and P proteins, was indispensable for the formation of IB-PM-like structures with low internal fluidity. These findings demonstrate that NiV employs specific protein–protein interactions to generate spatially and functionally distinct IBs, providing new insight into the molecular mechanisms governing viral RNA synthesis and particle formation.

**Impact Statement**

Nipah virus (NiV) forms two distinct types of inclusion bodies (IBs): cytosolic IBs, which serve as sites of viral RNA synthesis, and those beneath the plasma membrane (IB-PMs), which are involved in virion assembly. Using a plasmid-based system, we identified that dual interactions between the nucleocapsid protein and the phosphoprotein are essential for generating liquid-like cytosolic IBs, whereas the matrix protein drives the formation of IB-PM structures with low internal fluidity. These findings reveal how NiV coordinates specific viral protein interactions to establish spatially and functionally distinct organelles, providing new insights into the mechanisms linking viral RNA synthesis and particle formation.

## Introduction

Nipah virus (NiV), a member of the family *Paramyxoviridae* within the order Mononegavirales, is a highly pathogenic zoonotic virus that causes acute encephalitis in humans, with reported case fatality rates ranging from 40 to 70% [1] and classified as a biosafety level 4 agent. The viral genome is a non-segmented, negative-sense RNA of ~18 kb that encodes six structural proteins: the nucleocapsid (N), phospho (P), matrix (M), fusion (F), glyco (G) and large (L) proteins [2]. The N protein encapsidates the genomic RNA to form the viral ribonucleoprotein (vRNP) complex, which serves as the active template for transcription and genome replication, together with the polymerase complex, composed of the P and L proteins. The M protein bridges the vRNP and the surface glycoproteins (F and G) and is essential for the assembly and budding of infectious viral particles from the plasma membrane (PM) [3].

Upon infection, mononegaviruses form membrane-less organelles (MLO) known as inclusion bodies (IBs) in the cytoplasm [4]. These cytosolic IBs are generated through liquid–liquid phase separation (LLPS) and function as hubs for viral RNA synthesis. As in other mononegaviruses, such as measles virus, mumps virus and rabies virus, the formation of NiV IBs requires both the N and P proteins in the cell [5,8]. LLPS is driven by weak, multivalent interactions among proteins and nucleic acids and is often mediated by intrinsically disordered regions (IDRs) or multiple modular interaction domains within participating proteins [9]. Indeed, the NiV N and P proteins possess IDRs at their C- and N-termini, respectively, and previous studies have shown that at least the IDR of the P protein is involved in cytosolic IB formation [8].

Recently, another distinct type of IB, termed IB-PM, has been identified in NiV-infected cells [10]. IB-PMs form in close association with the PM and are spatially independent of the cytosolic IBs. The M protein localizes to IB-PMs, and its formation is abolished in cells infected with M-deficient NiV, which fails to produce virions. These findings suggest that IB-PMs represent the site of viral particle assembly. However, the molecular mechanisms underlying the formation and functional properties of IB-PMs remain poorly understood. In this study, we characterized the two distinct types of NiV IBs.

## Methods

### Cell culture and transfection

Vero (African green monkey kidney) [American Type Culture Collection (ATCC) CCL-81] and 293T (human kidney) [ATCC CRL-3216] cells were purchased from the ATCC (Manassa, VA, USA) and cultured in Dulbecco’s modified Eagle’s medium (FUJIFILM Wako Pure Chemical Corporation, Osaka, Japan) supplemented with penicillin (100 U ml^−1^), streptomycin (100 mg ml^−1^) and 10% FBS.

Cells were transfected with plasmid DNAs using TranslT-LT1 Transfection Reagent or TranslT-293 Transfection Reagent (for 293T cells) (Mirus/Takara Bio, Shiga, Japan) in accordance with the manufacturer’s instructions.

### Plasmids

The DNAs coding NiV N, P and M genes were artificially synthesized with optimized codons for humans and cloned into the pCAGGS vector for the expression of an N- or C-terminally FLAG-, HA- or Myc-tagged protein in addition to a non-tagged protein, resulting in the plasmids pCAGGS-NiV-N, pCAGGS-NiV-N-FLAG, pCAGGS-NiV-N_ΔC_, pCAGGS-NiV-N_ΔC_ -FLAG, pCAGGS-NiV-P, pCAGGS-NiV-P-HA, pCAGGS-NiV-P_ΔN_, pCAGGS-NiV-P_ΔN_-HA, pCAGGS-NiV-M and pCAGGS-Myc-NiV-M. In addition, the cDNA of the AcGFP gene was inserted into the 5′ terminal end of the P gene of pCAGGS-NiV-P and pCAGGS-NiV-P_ΔN_, resulting in the plasmids pCAGGS-AcGFP-NiV-P and pCAGGS-AcGFP-NiV-P_ΔN_, respectively.

### Antibodies

Anti-NiV-N rabbit polyclonal antibody (pAb) and anti-HA (3F10) rat and anti-Myc (My3) mouse monoclonal antibodies (mAbs) were purchased from Antibody System (Paris, France), Roche (Mannheim, Germany) and MBL (Tokyo, Japan), respectively. Anti-HA (HA11) mouse mAb and anti-HA rabbit pAb were purchased from BioLegend (San Diego, USA). Anti-FLAG (M2) mouse mAb and anti-FLAG rabbit pAb were purchased from Sigma (St. Louis, MO, USA).

### Immunoblotting and immunoprecipitation

For preparation of 293T cell extracts, cells were washed twice with cold PBS and then lysed in cell lysis buffer [20 mM Tris/HCl, pH 7.5, containing 135 mM NaCl, 10% glycerol, 1% Triton-X 100 and protease inhibitor cocktail (cOmplete: Roche)]. For immunoblotting, the cell lysate was boiled in SDS sample buffer and subjected to SDS-PAGE. The proteins were transferred to PVDF membranes (Millipore, Bedford, MA) and incubated with the appropriate antibodies. Each protein was visualized using SuperSignal West Femto Maximum Sensitivity Substrate (Thermo Fisher Scientific Inc., Waltham, MA) and detected using an Amersham ImageQuant 800 (Cytiva, Marlborough, MA, USA). For immunoprecipitation, the cell lysate was pre-cleaned with protein G-sepharose (Cytiva). Antibody-protein complexes were purified with protein G beads and washed with cell lysis buffer three times. After boiling in SDS sample buffer, the proteins were separated by SDS-PAGE and processed for immunoblotting. Preparation and immunoprecipitation of each cell extract were performed at least three times, and representative data are shown.

### Immunofluorescence microscopy

Vero cells were fixed in PBS containing 4% paraformaldehyde for 20 min at room temperature. The cells were permeabilized with PBS containing 0.2% Triton X-100 for 10 min, blocked with PBS containing 2% BSA for 30 min at room temperature and incubated with the indicated primary antibodies for 1 h at room temperature. Nuclei were stained with DAPI. Samples were examined under an LSM 900 confocal laser microscope (Carl Zeiss AG, Oberkochen, Germany) equipped with a Plan-Apochromat 63× objective lens. A series of consecutive images were obtained in the Z-stack from the field of view. The recorded images were examined and processed using Zen ver. 3.7 software. The experiments were performed at least three times, and representative data are shown.

### Fluorescence recovery after photobleaching

Vero cells cultured in a glass-bottom dish were transfected with pCAGGS-NiV-N, -P and -AcGFP-P, with/without pCAGGS-NiV-M. After 48 h, the cells were observed by an LSM 900 confocal laser-scanning microscope equipped with an STX Stage Top Incubator (Tokai Hit, Shizuoka, Japan) heated at 37 °C. After acquiring five images (1 s per frame), bleaching of a cytosolic IB- or an IB-PM-like structure was performed on a circular region using a 488 nm laser.

### Statistical analysis

Differences between groups were evaluated using unpaired Student’s t tests. Error bars indicate the standard deviations of triplicate measurements. Statistical significance was assumed at *P<0.01, **P<0.001.

## Results

### Dual-site interactions between NiV N and P proteins are essential for the formation of IB-like structures with liquid-like properties

**Fig. 1.**
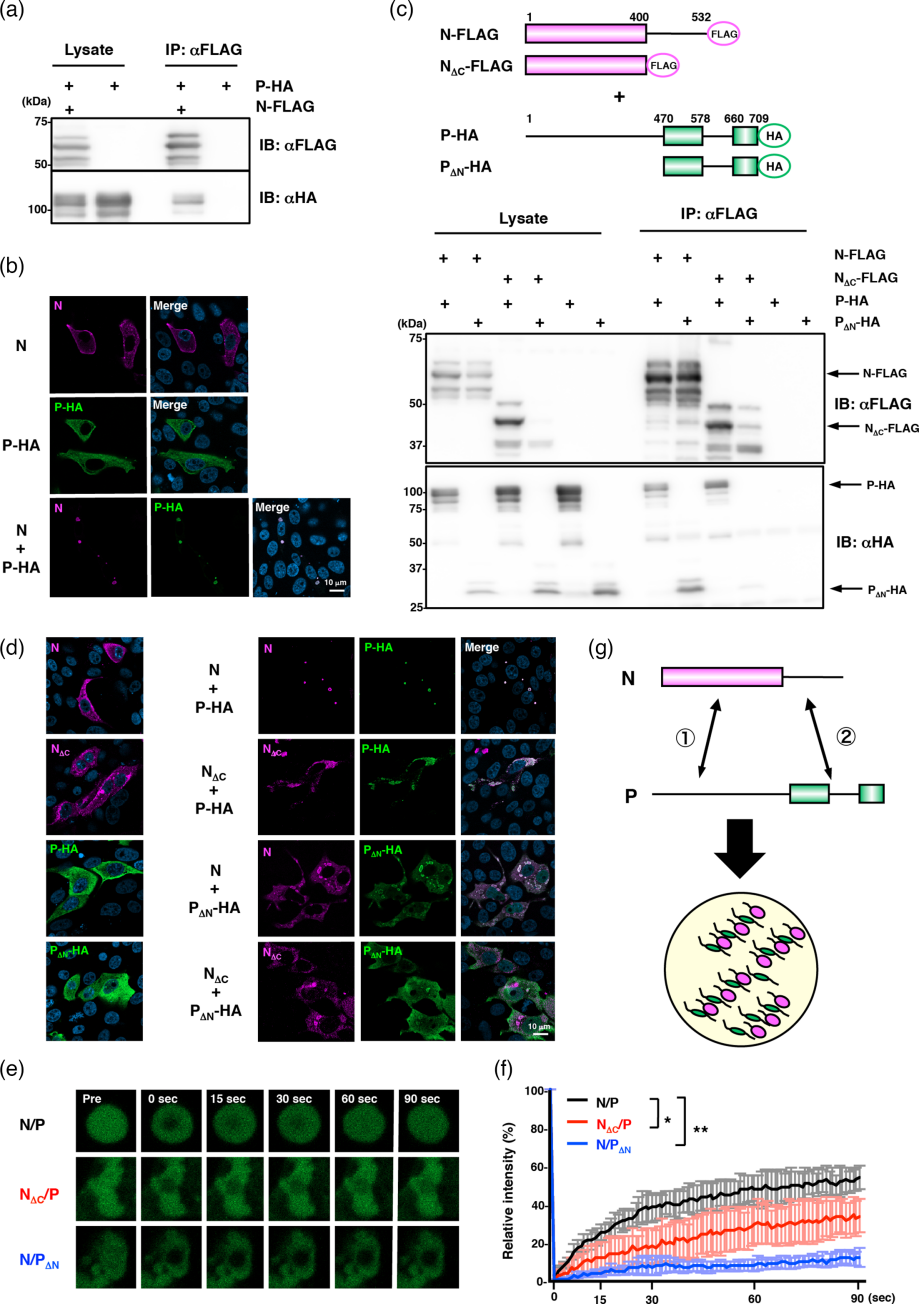
Dual-site interactions between NiV N and P proteins are essential for the formation of IB-like structures with liquid-like properties. (**a**) Immunoprecipitation assay showing the interaction of NiV N and P proteins. (**b**) Immunofluorescence assay showing the formation of cytosolic IB-like structure in Vero cells co-expressing NiV N and P proteins. (**c**) Immunoprecipitation assays showing that NiV N and P proteins interact via two distinct sites, located at the N- and C-terminal regions. The rectangles and the lines represent structured regions and IDRs, respectively. (**d**) Immunofluorescence assay showing the localization of NiV N, P-HA and their deletion mutants in Vero cells. (**e**) Fluorescence recovery images of AcGFP-P or -P_ΔN_ after photobleaching in Vero cells. (**f**) The normalized mean fluorescence recovery curve of AcGFP-P or -P_ΔN_. (**g**) Schematic image showing the interaction between NiV N and P proteins and the formation of cytosolic IB. The circled numbers 1 and 2 indicate the two interactions between the N and P proteins. The large circle represents a single cytosolic IB.

Consistent with a previous report [8], the NiV N protein and P protein interacted to form cytosolic IB-like structures (Fig. 1a, b). Then, to assess the contribution of IDRs of the N and P proteins to N–P interactions and the formation of cytosolic IB-like structures, we generated N and P mutants lacking the respective IDR regions. Immunoprecipitation assays revealed that the N_ΔC_ and P_ΔN_ mutants interacted with the full-length P and N proteins, respectively, whereas no interaction was detected between N_ΔC_ and P_ΔN_ themselves (Fig. 1c). Moreover, co-expression of the P protein, but not the P_ΔN_ protein, enhanced the expression level of N_ΔC_ protein. Immunofluorescence analysis further demonstrated that when the P_ΔN_ protein was expressed alone, it was diffusely distributed throughout the cytoplasm. In contrast, the N_ΔC_ protein was not only diffusely distributed but also formed localized foci. When the N protein was co-expressed with the P_ΔN_ protein, or the N_ΔC_ protein with the P protein, irregular cytoplasmic structures were formed, distinct from the spherical structures observed upon co-expression of the full-length N and P proteins. Within these irregular structures, the proteins colocalized. In contrast, colocalization was not observed when the N_ΔC_ and P_ΔN_ proteins were co-expressed (Fig. 1d).

We next evaluated the internal fluidity of cytosolic IB-like structures by fluorescence recovery after photobleaching (FRAP) analysis. As shown in Fig. 1(e, f), AcGFP-fused P protein exhibited rapid FRAP in Vero cells co-expressing full-length N and P proteins, demonstrating that these structures possess the characteristics of liquid organelles. In contrast, when the N_ΔC_ protein was co-expressed with the P protein in place of the full-length N protein, the recovery rate was markedly reduced. Moreover, little recovery of AcGFP-P_ΔN_ was observed in the foci formed by the P_ΔN_ and the N proteins. Collectively, these findings suggest that NiV N and P proteins interact via two distinct sites, located at the N- and C-terminal regions, and that both interactions are required for the formation of cytosolic IB-like structures with liquid-like properties (Fig. 1g).

### NiV M protein induces the formation of IB-PM-like structures with low internal fluidity through the interaction with N protein

During NiV infection, in addition to the cytosolic IBs that serve as sites of viral RNA synthesis, another type of structure, termed IB-PM, is formed at the cell periphery in proximity to the PM, where it is thought to function as a site for viral particle assembly and budding [10]. Whereas cytosolic IBs are formed by the N and P proteins, IB-PMs have been reported to additionally require the M protein. We therefore investigated the formation and characteristics of IB-PMs.

**Fig. 2.**
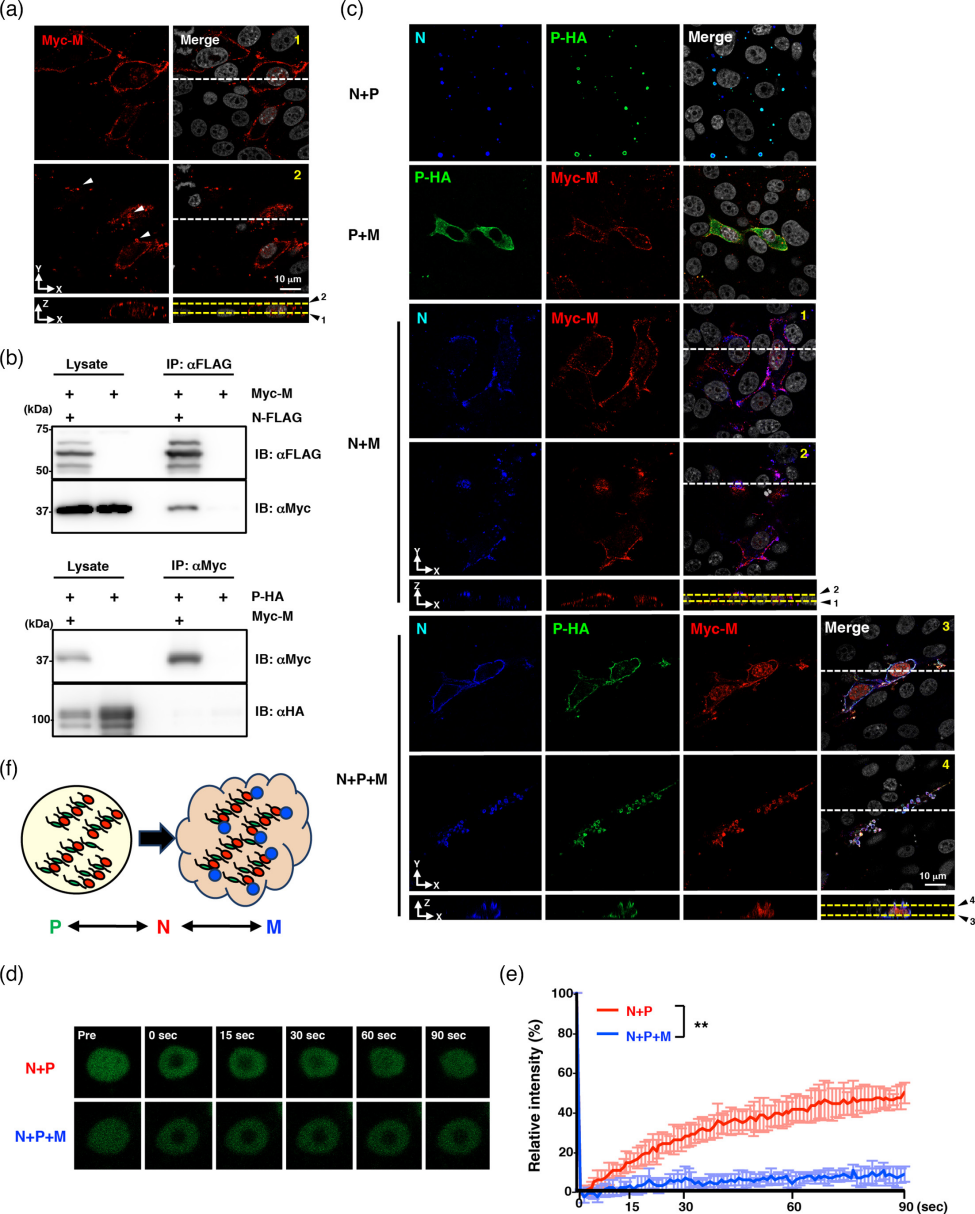
NiV M protein induces the formation of IB-PM-like structures with low internal fluidity through the interaction with N protein. (**a**) Immunofluorescence assay showing the intracellular localization of M protein expressed alone. The top and middle panels show XY images taken at the positions indicated by the horizontal lines labelled 1 and 2 in the bottom XZ orthogonal view, respectively. The bottom panels show XZ cross-sectional views obtained along the white dashed lines in the top and middle XY images. White arrowheads indicate clusters of M protein. (**b**) Immunoprecipitation assay showing the interaction of NiV M protein with the N but not the P protein. (**c**) Immunofluorescence assay showing the localization of the N, P-HA and/or Myc-M proteins in Vero cells. The panels 1–4 show XY images taken at the positions indicated by the horizontal lines labelled 1–4 in the XZ orthogonal view, respectively. (**d**) Fluorescence recovery images of AcGFP-P after photobleaching in Vero cells expressing the N and P proteins with/without the M protein. (**e**) The normalized mean fluorescence recovery curve of AcGFP-P in Vero cells expressing the N and P proteins with/without the M protein. (**f**) Schematic image showing the formation of cytosolic IB and IB-PM.

When expressed alone, the NiV M protein localized to the nucleus and cell periphery and additionally formed clusters in the cytoplasm opposite to the glass coverslip surface (Fig. 2a, line 2). Co-immunoprecipitation analysis revealed that the M protein interacted with the N protein, but not with the P protein (Fig. 2b). To further dissect the interactions, we examined the intracellular localization of viral proteins when two or all three were co-expressed in Vero cells. Co-expression of the P and M proteins did not alter their individual localization patterns, and no colocalization was detected. By contrast, when the N and M proteins were co-expressed, the localization of the M protein remained unchanged; however, unlike the diffuse cytoplasmic distribution observed for the N protein alone (Fig. 1b), the N protein partially colocalized with the M protein at the PM and in cytoplasmic clusters. Finally, co-expression of N, P and M proteins resulted in colocalization of all three proteins at the cell periphery and in cytoplasmic clusters located opposite to the coverslip surface and the structures that were considered to represent IB-PMs.

We next assessed the internal fluidity of IB-PM-like structures by FRAP analysis. As shown in Fig. 2(d, e), IB-PM-like structures exhibited markedly reduced internal fluidity compared with cytosolic IB-like structures. Collectively, these results suggest that the NiV M protein recruits the N and P proteins to cytoplasmic clusters through its interaction with the N protein, thereby inducing the formation of the IB-PMs with low internal fluidity that serve as sites for viral particle assembly and budding (Fig. 2f).

## Discussion

Cytosolic IBs, the sites of RNA synthesis in mononegaviruses, are known to be MLO formed through LLPS [4]. In this study, we extended the previous findings on the importance of the structural domain of the N protein and the IDRs of the P protein [8] and demonstrated that the interactions between the N- and C-terminal regions of the N and P proteins are both essential for the formation of cytosolic IB-like structures. When either of these two interactions was disrupted, cytoplasmic puncta were still formed but lacked the liquid-like properties. Notably, partial fluorescence recovery was observed when the P protein remained intact. Results from *in vitro* LLPS assays in several mononegaviruses have indicated that the P protein acts as a scaffold protein during LLPS [7,11, 12]. Consistent with this, our data suggest that the NiV P protein could play a more dominant role than the N protein in driving LLPS. Further investigation using *in vitro* reconstitution systems will be needed to elucidate the detailed mechanism of cytosolic NiV IB formation. In addition, the expression level of the N_ΔC_ protein was reduced when co-expressed with the P_ΔN_ protein. Since the P protein of paramyxoviruses is known to function as a chaperone for the N⁰ (RNA-free) [13], this result suggests that the NiV P protein contributes not only to the stabilization of the L protein but also to that of the N protein [14].

The formation of IB-PM is a characteristic feature of NiV infection and is thought to be involved in viral assembly and budding [10]. We found that co-expression of N, P and M proteins led to the formation of IB-PM-like structures at the cell periphery. These structures exhibited gel-like properties with low internal fluidity, distinct from the cytosolic IBs. Recent studies on Ebola virus (EBOV), another member of the order Mononegavirales, have reported that morphological and biophysical transitions of IBs occur during the late stages of infection, facilitating the transport of mature nucleocapsids to sites of virion assembly [15]. The cytosolic IBs and IB-PMs observed during NiV infection are likely to represent these phase changes of IB transition. In EBOV infection, the VP24 protein has been shown to mediate this process; in contrast, in NiV infection, the M protein appears to play a similar role. Thus, the NiV M protein not only interacts with both membrane proteins and the vRNP complex to trigger virion assembly but also promotes the transition from the RNA replication phase to the particle formation phase during infection. These findings suggest that the M protein functions as a key regulatory hub in the late stages of NiV propagation.

In mononegavirus infections, IBs serve as essential viral organelles that coordinate the major intracellular processes of viral propagation, from RNA synthesis to particle formation. This study provides new insights into the mechanisms underlying NiV IB formation and their biophysical properties. However, as our analyses were conducted using a plasmid-based expression system, future validation of these findings in the context of authentic NiV infection will be crucial for achieving a more comprehensive understanding of the viral growth mechanism.

## Abbreviations

ATCC: American type culture collection
EBOV: Ebola virus
FRAP: fluorescence recovery after photobleaching
IBs: inclusion bodies
IDRs: intrinsically disordered regions
LLPS: liquid–liquid phase separation
mAb: monoclonal antibody
MLO: membrane-less organelles
NiV: Nipah virus
pAb: polyclonal antibody
PM: plasma membrane
vRNP: viral ribonucleoprotein
